# An Ontological, Anthropological, and Psychoanalytic Perspective on Physician Burnout

**DOI:** 10.7759/cureus.34282

**Published:** 2023-01-27

**Authors:** Mayank Gupta, Edgar Chedrawy

**Affiliations:** 1 Psychiatry and Behavioral Sciences, Southwood Psychiatric Hospital, Pittsburgh, USA; 2 Surgery and Health Administration, Dalhousie University, Halifax, CAN

**Keywords:** philosophy, (hr 1667), dr. lorna breen health care provider protection act, mental wellness, wellness and resilience, physician suicide, professional burnout

## Abstract

Post-industrialization, societies have evolved with profound changes in ways of life. However, it was not until just recently that the overall impact of its deleterious and pernicious effects has been widely recognized, studied, and accepted. In the last few years, increased rates of stress and burnout (BO) not only affect clinicians, personally, but health care systems across the nation. The understanding of BO in the realm of current nosological limitations lacks context and is often unrecognized given the stigma associated with mental illness. However, the emerging data regarding this syndrome is highly concerning. Its impact on professionals and leaders across various disciplines is puzzling and underscores the nature and extent of the problem.

A search of PubMed, PsycINFO, Cochrane Library, and Google Scholar was conducted from the date of inception until June 2022 using the keywords Burnout”, “Mental Health”,” Physicians”, “Addiction”, “Health Care Workers”, “Anthropology”, “Psychopathology”, Philosophy”, “Wellness”. The search resulted in the identification of 135 articles; 56 articles met the inclusion criteria for the review.

Post-pandemic BO has reached distressing levels. BO remains poorly understood highly complex and has multifactorial etiology which is now included in the 11th Revision of the International Classification of Diseases (ICD-11) as an occupational phenomenon. Besides being linked with known factors such as long work hours, administrative burdens, and a lack of control over one's work environment there are many confounders. It is a serious issue that can have negative consequences for both the physician and the patient, and therefore mitigation strategies are needed. The awareness of these unmanifested conflicts, mindfulness training, limiting addictive habits, and efforts toward wellness may provide some alternative solutions. Lastly, developing a coherent philosophy may be useful for distress tolerance, dealing with ambiguity related to the profession, and paving the way to a more meaningful life.

## Introduction and background

In the 1960s the term "mid-life crisis" was often used to describe a transition of identity and roles leading to psychological unrest. During approximately the same time, the symptoms of physical and mental exhaustion were recognized as burnout (BO) in 1970. BO is distinct from depression as it is primarily related specifically to the workplace but remains a risk factor for depressive illness. BO is included in the 11th Revision ICD-11 as an occupational phenomenon and not classified as a medical condition. Healthcare professionals (HCP) have a long, often treacherous career path that can start as early as late adolescence and stretches until the mid-'30s. The decision to enter the healthcare field involves several aspects of critical thinking given that changes in the geopolitical landscape and regulatory requirements could affect overarching goals and objectives. These decisions could be considered informed but not informed enough since it is the prevailing notions of HCP's prestigious status in society, ultimate success, economic prosperity, and recognition in society that attracts many to the profession. The caveat of these scripts was that they stemmed from societal norms or beliefs, not from logic, reasoning, and empirical data.

Recognizing the issues around these conflicts requires the exploration of the human mind [1]. Since there are many who are at this juncture to accept this ambivalence or to continue as usual. On many occasions, the central and most compelling ontological questions are "who am I" and "why am I doing what I am doing?" [2]. The inquiries into these aspects of subjective intra-psychic conflicts may provide a contextual understanding of burnout.

These questions have a robust historic lineage, with detailed accounts in Greek philosophy from 2400 years ago. Socratic methods [3] are critical processes in the quest for truth that form a basic framework of today's evidence-based medicine. Plato expanded the dialectal methodology [4] to incorporate two alternative views in finding the truth and his epistemology laid the foundation of dualism [5]. Aristotle disagreed and instead believed that a soul is a form of the body [6]. In 623 B.C., Gautama Buddha was born as Prince Siddhartha in ancient India. He was thought to have been 29 years old when he began the quest for enlightenment, which was accomplished approximately six years later [7]. The famous American rock guitarist Jimi Hendrix (1942-1970) has been credited for the quote "Knowledge speaks, but wisdom listens [8]," which underscores the crux of the paradigm shift from the traditionalist's framework. Towards the end of the 19th Century, German philosopher Karl Robert Eduard Hartmann authored a highly influential book *Philosophy of the Unconscious* (1869) [9]. These were foundational thoughts that took off with the seminal psychoanalytic theories of Sigmond Freud, and thereby word "ego" came into existence. When one challenges the self, it often discomforts the ego. In a metaphorical sense, the ego's primal function is to keep one comfortable at all costs. In his famous book *Civilization and Its Discontents*, Freud also wrote that conformity with the rules is a primary reason for repression [10]. Carl Jung argued that these unconscious desires or drives need to be integrated and not repressed for fuller internal experiences [11]. The overview of these complex historical contexts provides an understanding of the human quest among many overlapping disciplines.

## Review

Methods

A comprehensive search of several databases from the date of inception to the date of the search was conducted. The databases include PubMed, PsychINFO, Cochrane Library, and Google Scholar as well as the databases of ongoing clinical trials through clinicaltrials.gov. The search was designed using controlled vocabulary and keywords "Burnout", "Mental Health", "Physicians", "Addiction", "Health Care Workers", "Anthropology", "Psychopathology", "Philosophy", and "Wellness". It was performed in all languages and was limited to human subjects. We also performed a manual search. The inclusion criteria were any published material on burnout with links to physicians. There were 135 published materials after the removal of duplicates. After reviewing the abstract, only 30 studies met our inclusion criteria, and 26 other studies were added manually after reverse citations were reviewed to update the material.

Results and discussion

Epidemiological Trends

As per current data, 400 physicians die by suicide each year. The rate of physicians and nurses dying by suicide is two times the general population's rate. BO increases the risk of medical errors by 200%. The Triple Aim approach to improving healthcare systems was initially introduced in 2008. Given that BO affects quality, the Quadruple Aim approach was introduced in 2014 [12]. These expand upon the Institute for Healthcare Improvement's (IHI) Triple Aim goals of improving the health of populations, enhancing the patient experience of care, and reducing costs. It branded a self-evident truth that was not previously formally recognized in healthcare: improving the clinical experience for healthcare workers as a prerequisite for the first three goals. Acknowledgment of the Fourth Aim is the only path to a sustainable healthcare system workforce [13].

In the United States on March 18th, 2022, the Dr. Lorna Breen Health Care Provider Protection Act (HR 1667) aimed to reduce and prevent suicide, BO, and mental and behavioral health conditions among healthcare professionals [14]. Additionally, a recent high-profile lawsuit in Tennessee that resulted in a felony conviction of a nurse for death due to medical error has raised serious concerns among critical care nurses [15]. The Statements from the American Nurses Association, the American Association of Critical-Care Nurses, and the National Medical Association unanimously stated that it set a "dangerous precedent". These trends point toward an unrecognized epidemic of chronic healthcare fatigue and exhaustion.

Origins and Phenomenology of Burnout

The American psychologist Herbert Freudenberger coined the term BO in the 1970s [16]. According to World Health Organization's (WHO) International Disease Classification (ICD-11), burnout is characterized by "feelings of energy depletion or exhaustion, increased mental distance from one's job, or feelings of negativism or cynicism related to one's job; and reduced professional efficacy."

It is notable that the nosology of mental disorders in recent times has moved towards the categorical and dimensional presence of symptoms. In earlier psychiatric traditions, there was a wider phenomenological recognition of the clinical context behind symptoms for understanding, diagnosis, and treatment [17]. The German philosopher G. Hegel describes phenomenology as a philosophical (*philosophischen*) and scientific (*Wissenschaftliche*) study of phenomena, as a method of understanding a logical, ontological, and metaphysical reflection of human thinking [18].

The debates about the origin of symptoms started in the last two centuries. The earlier writing of Sigmond Freud describes symptoms as follows: "A symptom is a sign of, and a substitute for, an instinctual satisfaction which has remained in abeyance; it is a consequence of the process of repression" [19-20]. In simple terms, symptoms have meanings of unmanifested and complex psychic processes. William Abel Caudill (1920-1974), the founder of medical anthropology, conducted many field trials including his groundbreaking work at Yale Psychiatric Institute, and authored the book *The Psychiatric Hospital as a Small Society* [21]. Caudill identified a myriad of feelings among different staff members, and communication problems were attributed to the higher specialization of medical positions. Since 1963, empirical research stemming from the disciplines of medical anthropology has been instrumental in understanding the socio-cultural aspect of workings within healthcare.

BO as a construct is a subjective experience but can be measured using the Maslach Burnout Inventory [22]. There has been an undisputed neurobiological and molecular base for these syndromes; however, we have explored ontological, anthropological, and psychological models to give contextual understandings of these phenomena. The mindful self-awareness of these powerful processes when applied in conjunction with scientific methods has utility in finding solutions [23].

Anthropological studies suggest humans' primal needs are to feel safe and secure, which translates into centuries of human struggles and discoveries to be where we are [24]. In typical developmental stages of life, the teenage years are all about developing identity. The 20s include university education, getting their first jobs, and trying to get their foot in the door of their dream as per the script laid out in the developmental years [25]. Now it's widely accepted that physical strength peaks at the age of 33 and mental growth spikes at 28. So, by the time healthcare professionals reach their mid-30s, they have progressed in their careers and attained a position many people desire. It's inner turbulence that people struggle to find a reason for, and many attempts to overcome it by actively engaging in social media, yoga, and cultural groups. But for many, these are mostly temporary solutions and often lead back to square one when it comes to figuring things out. On many occasions, folks gravitate towards instant gratification, which is when the role of dopamine comes into play. Addiction research overwhelmingly reports that alcohol, sugar, sex, and money energize common dopaminergic reward pathways in the brain. The vulnerable states are known to put many at higher risk of addiction. Why is this feeling more common during the late 30s and 40s? Let's delve deeper into the plausible explanations for these stages in the subsequent sections.

Alternative Explanations

In the last decade, there has been cross-cultural empirical research that has linked multiple variables with burnout. A few of them include long working hours, sleep deprivation chronic fatigue of circadian rhythm disruption, second victim syndrome (SVS), electronic health records, long work hours, and substantial educational debt [26]. Besides that, medicolegal risk, workplace harassment, breakdown in personal relationships like divorce, and deaths are a few among many contributing to BO [27].

There are many with higher risks of burnout, with clear gender differences given that females have higher stress often due to additional work caring for children and families [28]. There are also individual variations in the degrees of resilience that also contribute to the perpetuating risk factors. BO initially presents with a mixed range of symptoms and may develop into a mental health disorder with serious impairments in functioning. But many other confounding variables, which are often counterintuitive, are worth further exploration. 

The mere acknowledgment of the existence of the human mind may help us identify the elephant in the room. On many occasions the mind is indoctrinated by rigid societal beliefs in childhood before its ability to reason is developed, and therefore conformity perpetuates without questioning. The mysteries behind the hallmarks of adolescence like risk-taking, sensation-seeking, and dysregulated emotions have puzzled scientific communities. This enigma has been unraveled by groundbreaking work about the delayed development of the prefrontal cortex (the highest seat of executive functioning) [29]. It is now widely accepted that the brain maturation process is only completed at ages 25-26 years of life. These developmental gaps may affect the ability to ask difficult questions, distant from the comfort of conformity. Whenever we try to step out of the realms of our belief system, it becomes overwhelming. We may ignore our desires and conform to convention while the unconscious mind will keep reminding us of these hidden expectations and desires. These often come back in the form of nightmares [30], anxiety, irritability, and physical pain. The incongruence with self is manifested when one intends to seek pursuits and gets in conflict with societal values. This invisible dissonance is often intolerable but unlike physical pain, this mental agony shows itself in the form of headaches, sleeplessness, relationship issues, and irritability which most people fail to effectively deal with. These states could lead to heightened vulnerability for many impulsive decisions and adds to the risks for mental health conditions. It seems like although the body physically plateaus at this stage, the mind is rapidly evolving.

The Changing Landscape of Friends and Family

The 40s is a time when relationships and roles begin changing as members of this age demographic aren't as agile and healthy as they used to be. At the same time, the new generation is now rebellious as a result of underlying rapid brain changes and pruning [31]. They have a unique way of looking at the world, a view shaped vastly by the social and cultural environments outside the four walls of the home. It's made the world smaller and children of the current generation more open to the blending of cultures and more liberal views. This leads to more questioning of traditional values which further accentuates the anxiety. A divorce or the death of someone of the same age could also be the reason for feeling unsettled at this age. Going out into the world to find love again seems to give you the feeling that an individual might be a little out of practice at it while losing a peer could lead to questions and anxiety about one's health.

In his seminal work, an influential psychiatrist Dr. Harry Stack Sullivan wrote that preadolescents (10-13 years of age) enter a period of chumship to find someone and share their innermost thoughts and feelings. During this process, they can explore and reveal both positive & negative aspects of themselves without fear of being judged in an environment of total acceptance [32]. This is critical for developing personality and accepting many negative self-evaluations and others' points of view. The chumship is symbolic of a period wherein a friend there is a total acceptance and consensual validation of aspects of being human. It's so often that in the polarized world we live in those minimal disagreements could lead to perpetual avoidance and self-inflicted isolation and loneliness to protect one's vulnerabilities. The chumship of mid-adulthood is more sophisticated, given that personalities are fully formed, and the world is viewed from multiple vantage points. Therefore, if one operates with sheer dominance; then it may push friends away.

Aging, Social Life and Habits

There are many reports of higher longevity in women as compared to men. Research shows that women often have better subjective self-awareness of their body's functions since their teenage years because of their menstrual cycle; they understand hormonal changes better with every passing month [33]. Men in comparison have a later and slower transition into aging with a shortening of the telomeres [34]. Likewise, there are emerging findings about pregnancy and childbirth dramatically accelerating aging in women linking shorter telomeres to the mechanisms behind earlier theories of costs of reproduction' (CoR) [35].

Conventionally, our social circles form a good, healthy part of our lives. But with the advent of social media, socializing has changed in the last 15 years. Face-to-face contact and subtle non-verbal communication also have a positive effect. But in the last decade, there's been a constant race to put your best foot forward by editing out imperfections. It is widely known that people connect more with their vulnerabilities than their strengths [36]. So, when you constantly see your peer group at their edited best, it makes you question your life choices. This leads to a less strong bond than you would've had with someone who showed you both sides of their seemingly perfect life. Among the sea of ideas and beliefs, people are hyper-sensitive toward criticism and they have less tolerance towards others. As a result, people are more isolated and don't enjoy those robust and healthy life-long friendships, which take a toll on them.

The pleasure-seeking behaviors are linked to striatal dopaminergic neurons of the reward center. Many self-medicate during dysphoric states with activities that potentiate these circuits [37]. The transition from use to abuse could be subtle and as one idiom summarizes it well "it's a temporary solution which itself becomes a big problem". The loss of control over these behaviors could lead to obesity, alcoholism, internet addiction, and gambling. Experts say adults should limit screen time outside of work to less than two hours per day.

Mitigation Strategies

Socrates, whose ideas have influenced modern Western philosophy, is famously quoted as having said that "the unexamined life, for a human, is not worth living" [38] "The unexamined life" means people have no questions, they never question life, they don't want to know about the truth, and they don't know who they are. Many skilled professionals excel in the workplace but find it hard to derive purpose in what they do. Without this, work could be a monotonous circular exercise that begets confusion, frustration, and boredom. Nowadays, boredom has readily available fillers with highly addictive potential like round-the-clock TV, social media, shopping, and other ways to distract. On the contrary, if one finds meaning and purpose in work or outside work, the experience would be different.

So, what could be the potential solutions? The solution(s) must be part of a comprehensive approach to building wellness or resilience for healthcare workers (HCWs). The overarching goals include reframing or developing newer priorities or measures of success and addressing the stigma around self-care. Firstly, establish a culture of recognition of these issues. Research supports that organizations with high-recognition cultures benefit from less turnover and better performance since informed work environments breed peer support and integration. Second, HCWs should be educated and encouraged to have real weekends and holidays. Burnout is likely when there aren't given enough time to disconnect, rest, and focus on other aspects of life. Lastly, employers must expand wellness programs and benefits: Family leaves, "flexible work options", office health and wellness programs, paid time off for "mental health" or recuperation days, and stress management training for employees to preempt burnout. A few companies, for example, provide free yoga and meditation classes, on-site fitness centers, nutritious food options, and financial incentives for healthy living [39]. Organizations can and should play a more active role in preventing burnout. Besides organizational measures, the HCWs may consider some of the following strategies.

The initial steps in the process would require reconciling one's overarching philosophy of existence with a goal of systematic expansion and self-appraisals utilizing dialectical questioning in finding meaning and purpose. The practice of mindfulness, self-awareness, and time without distractions, including screens, to tune in to the intrapsychic space has the potential for self-exploration. In his seminal work, Donald Winnicott (1958) [40] wrote "the capacity to be alone is a highly sophisticated phenomenon closely related to emotional maturity". Although his work was primarily focused on children, it has many symbolic meanings like "Being able to enjoy being alone along with another person who is also alone is an experience of health". To make things easier, an expert may help learn how to spend time tuning in with yourself. It could be self-exploration or guided with the help of a trained therapist. It's important to note, that therapy does not mean getting advice or direct answers to problems in your conscious awareness but it's a process of uncovering the self and exploring hidden unexplored repressed materials.

A smaller and more frequently healthy portion and avoiding sugar for a month are simpler lifestyle modifications. In the last five years, evidence suggests that chronically irritated leaky gut is a risk factor for many neuropsychiatric conditions [41]. This is a highly modifiable lifestyle one may adopt and efforts to restore gut health could alleviate many symptoms. The 10,000 steps every day and regular stretching are practical measures to stay fit [42]. For instance, practicing slow and controlled breathing seven times a minute for five minutes using the abdomen muscles (not the chest) is recommended every hour, five times a day. This practice has the potential to activate the frontal cortex [43]. A few experts believe if the abdominal muscles are too tight, it may restrict deep diaphragmatic breathing and affect overall health [44]. Thanks to the urban, highly mechanized lifestyles we continue to stoop and slouch. Neck, back, temporomandibular (TMJ) [45], and shoulder pains are common modern lifestyle conditions associated with poor posture. Stretching for five minutes at least three times a day would provide nutrition to those avascular tendons and ligaments. These structures get nutrients with diffusion since they don't have a direct blood supply [46-47].

Dependency on substances provides a transient sense of relief, not long-term. In simple terms, addiction could be theorized [48] as a loss of control. Excessive caffeine is also linked to anxiety, so cutting it down could be an easy solution [49]. Sleep deprivation may have serious long-term effects on overall well-being [50]. In many cases, this is modifiable using basic sleep hygiene. In refractory cases, a complete assessment and consulting specialist to rule out common but highly underdiagnosed obstructive sleep apnea may be of value.

Friendships are considered associated with a state of Aristotelian eudaimonia [51]; sharing and appreciating limitations and vulnerabilities are critical elements that foster closeness. The self-disclosure is positively linked with intimacy, solidarity, commitment, and mutual satisfaction in relationships [52]. A counterintuitive finding by Canadian and American psychologists published in 2008 reports that spending money on others even as little as $5 may promote overall well-being [53]. This study has a deep psychological context that may be explored individually with small giving. Lastly, Freud said "Love and work are the cornerstones of our humanness" and Winnicott emphasized the role of play in both the core development of children and its intrinsic therapeutic value in self-healing [54]. The interplay between these concepts could guide and help balance out disequilibrium. And the aesthetics or various systems of art is an individual's pathway to connect, explore and express. For example, Hegel regards poetry as the "most perfect art" (PKÄ, 197), and the "most unrestricted of the arts" (Aesthetics, 2: 626) [55]. Likewise, Heidegger writes, "Poetry is what first brings man onto the earth, making him belong to it, and thus brings him into dwelling" [56]. Table 1 summarizes a few of these strategies. 

**Table 1:** Strategies to reduce burnout.

	Summary of Strategies
Time with Mind	Retreat with a focus on self and the capacity to be alone without a screen
Gut-Brain Axis	Reduce sugar and self-educate about dietary lifestyle modifications
Exercise	Measure activity, stretch before exercise and learn slow breathing techniques
Posture	Ergonomic workspace with active stretching
Addiction	Cut down on sugar, alcohol, caffeine, technologies ,etc.
Sleep	Sleep hygiene and reduce deprivation
Hobbies	Any forms of play, music, and poetry
Relationships	Foster long-term relationships when one may share vulnerabilities
Learn	Lifelong learning above and beyond professional fields and efforts to develop a coherent philosophy

Limitations

There are limitations to this review. While the phenomenon of BO continues to evolve in the scientific literature, the current review is a broader overview of the topic related to these areas of research. The subjective selection bias and non-protocol-based selection are a few of the many implicit biases associated with the narrative literature review. There are low and insufficient empirical studies associated with BO and our article encompasses an overarching review of BO which is descriptive in nature. These phenomenological descriptions and psychoanalytical concepts lack objective underpinnings; however, they provide a unique perspective for readers and develop models for future research.

## Conclusions

Burnout among professionals is highly prevalent and a spike in the trends is observed across disciplines. It's also recognized among academic associations, professional organizations, and the state, and federal levels, underscoring the need for collaborative efforts to curb its deleterious effects. This recognition is a step in the right direction, but individualized and contextual alternative explanations are required to help many understand the nature of the issue. The awareness and acceptance itself have the potential to mitigate some effects of burnout. However, nuanced, balanced, and meaningful communication is required to engage and address these blind spots. Stress is inevitable in the workplace but it doesn't have to be pervasive and pernicious, therefore awareness and targeted interventions have a significant role. Lastly, besides work, leisure, and rest, there is a need to develop a coherent philosophy to navigate the challenges, dilemmas, and ambivalence that are an implicit part of human life. These explorations, self-examination, and inquiries are options when many intuitive and known solutions are ineffective. The goal of these quests is lasting personal growth with self-driven appraisals to live a meaningful and authentic life.
